# Acute Obturator Externus Injury in Professional Soccer Players: A Case Series

**DOI:** 10.3390/medicina58091145

**Published:** 2022-08-23

**Authors:** Hye Chang Rhim, Ashley E. Gureck, Ki-Mo Jang

**Affiliations:** 1 Department of Physical Medicine and Rehabilitation, Harvard Medical School, Boston, MA 02115, USA; 2 Department of Orthopedic Surgery, Anam Hospital, Korea University College of Medicine, Seoul 02841, Korea

**Keywords:** obturator externus, soccer injury, muscle injury

## Abstract

When patients present with hip or groin pain, proximal quadriceps or adductor injuries are often initially suspected. In this case report, however, we present three cases of professional soccer players who were found to have obturator externus injury. A 30-year-old player and a 24-year-old player complained of pain in the left side after long distance shooting during an in-season training session and a match, respectively. Another 24-year-old player complained of pain in the right side after long distance passing during a preseason training session. On physical examination, active hip external rotation and passive hip internal rotation and extension elicited pain in all three players. All three players underwent magnetic resonance imaging (MRI) which found obturator externus grade II injuries for two players and grade I injury for one player. Rehabilitation protocols included relative rest, cryotherapy, and electrotherapy over a period of one week. All patients were able to return to play after 10 days. Correct identification of obturator externus injury afforded our players a favorable prognosis and a relatively quick return-to-sport compared with quadricep or adductor injury.

## 1. Introduction

Hip and groin pain accounts for an estimated 4–19% of all injuries in male professional soccer players [1] and may result in time loss for one in five players each season [2]. In an effort to mitigate the complexity involved with diagnosing and classifying injuries to the various structures that make up the hip and groin, the Doha agreement meeting was held in 2014. As a result, groin pain was classified into three major categories: (1) defined clinical entities for groin pain which include adductor-related, iliopsoas-related, inguinal-related, and pubic-related groin pain (2) hip-related groin pain and (3) other causes of groin pain in athletes [1]. According to Mosler et al., adductor-related groin pain was the most common type followed by iliopsoas- and pubic-related groin pain [2]. Although rare compared to adductor, iliopsoas, and pubic-related groin pain, injury to the deep external rotators of the hip may present with anterior hip or groin pain.

The deep external rotators of the Hip include piriformis, gemelli, quadratus femoris, and obturators. These muscles have complex origins and reside deep within the pelvis making them challenging to isolate on physical examination. Because these muscles are either under-reported to be injured or misdiagnosed as hamstring, adductor, or quadriceps injuries [3], there have been limited studies describing injuries to these muscles. Understanding the nature of these injuries would be crucial for clinicians, especially those dealing with athletes, to devise diagnostic and treatment plans as well as to guide their return to play. In light of such importance, the purpose of this case report was to present three professional soccer players who sustained obturator externus muscle injuries and describe their injury characteristics.

## 2. Presentation of Cases

Between March 2021 and February 2022, three professional male soccer players recruited to the South Korea national team, presented with acute onset of groin and anterior hip pain. The pain was dull and achy. A 30-year-old player (Player A, 175 cm/73 kg, right leg dominant) and a 24-year-old player (Player B, 173 cm/68 kg, left leg dominant) complained of pain in the left side after long distance shooting during an in-season training session and a match, respectively. Another 24-year-old (Player C, 168 cm/65 kg, right leg dominant) player complained of pain in the right side after long distance passing during a preseason training session. All these players denied any numbness or weakness and radiating pain down to the legs. On inspection, there was no obvious bruising, ecchymosis, edema, or swelling. On palpation, there was no obvious tenderness. Active hip external rotation and passive hip internal rotation and extension elicited pain in all three players. These players were able to walk without limping.

**Figure 1:**
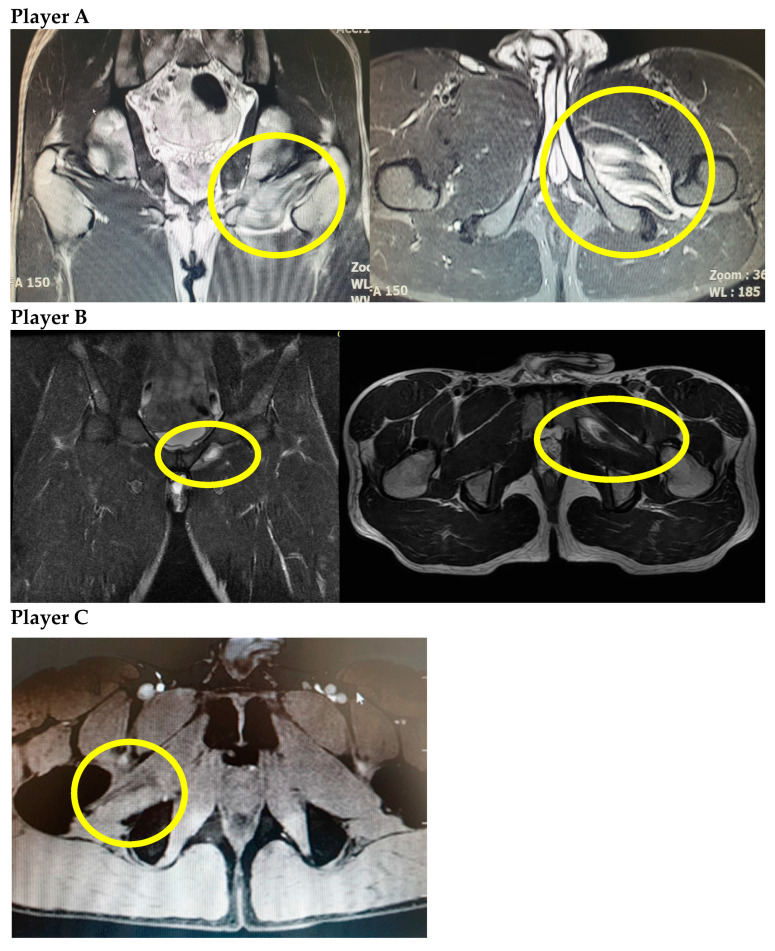
MRI of obturator externus injury in three professional soccer players. **Player A**: Hip joint MRI demonstrates grade II injury of left obturator externus muscle, coronal image (left) and axial image (right). **Player B**: Hip joint MRI demonstrates grade II injury of left obturator externus muscle, coronal image (left) and axial image (right). **Player C**: Hip joint MRI demonstrates grade I injury of right obturator externus muscle.

Ultrasound imaging was done for Player C but could not locate the source of pain. All three players underwent magnetic resonance imaging (MRI), and the injuries were classified as grade I (strain) if the injury consisted of a minor degree of microscopic tearing without macroscopic muscle fiber discontinuity and grade II (partial tear) if the injury showed an incomplete disruption of muscle fibers with edema and hemorrhage within the muscle or at the muscle-tendon junction [4]. Players A and B demonstrated obturator externus grade II injuries, while Player C sustained grade I injury (Figure 1). Rehabilitation protocols included relative rest, cryotherapy, and electrotherapy over a period of one week. During this period, these players continued core and lower extremity exercises as tolerated without invoking pain. All patients were able to return to play after 10 days. All three players provided informed consent for this report.

## 3. Discussion

Though proximal quadriceps or adductor injuries are often initially suspected [5,6], this case report highlights the importance of considering obturator externus injury in athletes presenting with diffuse hip or groin pain and a suspicious mechanism of injury.

The obturator externus originates from the external bony margin of the obturator foramen with a cylindrical tendon passing under the femoral neck and inserting in the trochanteric fossa. The primary function of the obturator externus is to externally rotate when the hip is in neutral position and flexed at 90 degrees, while the secondary action is to adduct when the hip is in flexion [7].

Because there is no established physical examination to differentiate obturator externus injury from other potentially more common injuries, a high index of clinical suspicion is warranted. For instance, Wong-On et al. showed that most of the patients had pain during passive flexion, adduction, and internal rotation of the hip [8], while in our three patients, active hip external rotation and passive hip internal rotation and extension elicited pain.

The largest case series to date identified potential mechanisms for obturator injuries in soccer players as follows: (1) unstable change of direction trying to control the ball (2) anterior or lateral hip slide in an unstable position (3) repetitive ball kicking and (4) kicking the ball in an unstable position [8]. Based on our observation of three players, we speculate that long distance shooting or passing may potentially lead to obturator externus injury by way of sudden, powerful muscle contraction with hip flexion and adduction. This observation is consistent with a previous cadaver study which demonstrated that maximal strength of the obturator externus derived from an extended position towards flexion and adduction [9]. Therefore, this biomechanical factor should be considered for exercise progression during rehabilitation [10].

Due to the deep location of this muscle, ultrasound cannot reliably identify this injury. In our case, MRI was essential to identify the location and degree of injury. Correct identification of obturator externus injury afforded our players a favorable prognosis. Just as no direct relationship was seen between MRI finding and the return to play time [8], our three players returned to play after 10 days despite differing degrees of injury. This return to play time was also similar to the mean reported in the previous study [8] with another case series reporting a maximum of 21 days. Based on our findings and previous case series, this return to play time is in general faster than what is expected for other short muscles [11,12] and other biarticular muscle injuries around the hip such as rectus femoris and hamstrings [13].

Our findings are meant to aid clinicians in understanding the mechanism of obturator externus injury and therefore consider this injury in cases of athletes presenting with acute hip or groin pain. Common in-office techniques including thorough musculoskeletal physical exam or ultrasound may not reliably identify obturator externus injury due to its deep location, inability to directly palpate, and difficulty to isolate. Therefore, advanced imaging with MRI should be considered in cases where injury is suspected. Although obturator externus injury is apparently relatively infrequent, maintaining a high degree of clinical suspicion may allow for better detection and therefore a more accurate understanding of prognosis and rehabilitation recommendations. Patients may be reassured by the relatively benign nature of this injury and ability to treat with conservative modalities.

We acknowledge that there are several limitations in our report. First, given that our cases were professional athletes, our findings may not be applied to the general population or amateur athletes. Second, while our three cases did not have ongoing pain or recurrence of injury at one year, we were limited by a relatively short follow-up period in understanding the nature of this injury on a longer-term basis.

Future studies should aim to identify the demographic, biomechanical, and sport-specific factors that may contribute to obturator externus injury. Such identification is an important step forward for informing rehabilitation and injury prevention strategies. This can be especially important for high-level athletes whose careers may be adversely affected due to lost play or practice time. Research should also seek to determine whether such an injury has the tendency to recur, or have any long-term repercussions, such as abnormal recruitment of synergistic hip muscles with downstream biomechanical effects that may contribute to subacute to chronic hip pain.

## 4. Conclusions

Obturator externus tear can present with acute onset of groin and anterior hip pain in professional male soccer players after long-distance shooting or passing. Correctly recognizing obturator externus injury allows for conservative management and a relatively short recovery period.

## Data Availability

Not applicable.

## References

[1] Weir A., Brukner P., Delahunt E., Ekstrand J., Griffin D., Khan K.M., Lovell G., Meyers W.C., Muschaweck U., Orchard J. (2015). Doha agreement meeting on terminology and definitions in groin pain in athletes. Br. J. Sports Med..

[2] Mosler A.B., Weir A., Eirale C., Farooq A., Thorborg K., Whiteley R.J., Hӧlmich P., Crossley K.M. (2018). Epidemiology of time loss groin injuries in a men’s professional football league: A 2-year prospective study of 17 clubs and 606 players. Br. J. Sports Med..

[3] Khodaee M., Jones D., Spittler J. (2015). Obturator Internus and Obturator Externus Strain in a High School Quarterback. Asian J. Sports Med..

[4] Dimmick S., Rehnitz C., Weber M.-A., Linklater J.M. (2013). MRI of muscle injuries. Magnetic Resonance Imaging of the Skeletal Musculature.

[5] Valente H.G., Marques F.O., Souza L.D.S.D., Abib R.T., Ribeiro D.C. (2011). Injury of the external obturator muscle in professional soccer athletes. Rev. Bras. Med. Esporte.

[6] Sirico F., Palermi S., Massa B., Corrado B. (2020). Tendinopathies of the hip and pelvis in athletes: A narrative review. J. Hum. Sports Exerc..

[7] Gudena R., Alzahrani A., Railton P., Powell J., Ganz R. (2015). The Anatomy and Function of the Obturator Externus. HIP Int..

[8] Wong-On M., Turmo-Garuz A., Arriaza R., De Suso J.M.G., Til-Perez L., Yanguas-Leite X., Diaz-Cueli D., Gasol-Santa X. (2018). Injuries of the obturator muscles in professional soccer players. Knee Surg. Sports Traumatol. Arthrosc..

[9] Vaarbakken K., Steen H., Samuelsen G., Dahl H.A., Leergaard T.B., Stuge B. (2015). Primary functions of the quadratus femoris and obturator externus muscles indicated from lengths and moment arms measured in mobilized cadavers. Clin. Biomech..

[10] Palermi S., Massa B., Vecchiato M., Mazza F., De Blasiis P., Romano A.M., Di Salvatore M.G., Della Valle E., Tarantino D., Ruosi C. (2021). Indirect Structural Muscle Injuries of Lower Limb: Rehabilitation and Therapeutic Exercise. J. Funct. Morphol. Kinesiol..

[11] Cass S.P. (2015). Piriformis Syndrome: A cause of nondiscogenic sciatica. Curr. Sports Med. Rep..

[12] Willick S.E., Lazarus M., Press J.M. (2002). Quadratus Femoris Strain. Clin. J. Sport Med..

[13] Brukner P.D., Connell D. (2016). ‘Serious thigh muscle strains’: Beware the intramuscular tendon which plays an important role in difficult hamstring and quadriceps muscle strains. Br. J. Sports Med..

